# Exploring homology detection via k-means clustering of proteins embedded with a large language model

**DOI:** 10.1093/bioinformatics/btaf472

**Published:** 2025-08-26

**Authors:** Thomas Minotto, Antoine Claessens, Thomas D Otto

**Affiliations:** LPHI, CNRS, University of Montpellier, Montpellier 34095, France; IMAG, CNRS, University of Montpellier, Montpellier 34095, France; LPHI, CNRS, University of Montpellier, Montpellier 34095, France; LPHI, CNRS, University of Montpellier, Montpellier 34095, France; School of Infection and Immunity, University of Glasgow, Glasgow G12 8TA, United Kingdom

## Abstract

**Motivation:**

Inferring protein homology from sequence information is essential for understanding species evolution and enabling functional annotation transfer. Besides similarity-based methods, several machine learning approaches have been developed using various ways of representing protein data.

**Results:**

Here, we represent proteins with a biologically oriented large language model and apply k-means clustering to the embedded data to extract homology relationships. Although our approach lacks the sensitivity of other tools, we obtain better precision for the detection of n:m orthologs. Furthermore, we successfully reconstruct full orthologous groups from scratch, highlighting the growing potential of using large language models in combination with clustering algorithms for the analysis of protein data.

**Availability and implementation:**

Datasets are available on OrthoMCL-DB as indicated in the Methods. Source code is available on GitHub at https://github.com/ThomasGTHB/OrthoLM and Zenodo at https://doi.org/10.5281/zenodo.16640170.

## 1 Introduction

Deciphering protein function is a fundamental task in biology. However, experimental approaches to determine protein function are often resource-intensive, costly, and difficult to scale to entire genomes. To address this challenge, computational methods have been developed to infer functional information directly from amino acid sequences. These methods leverage sequence comparisons, assuming that similar sequences are likely to have similar functions. Such comparisons can be applied to individual protein pairs or used to group proteins based on shared similarities without restrictions on group sizes. When sequence similarity arises from shared evolutionary origins, the proteins are termed homologs. Homologs between different species, known as orthologs, are particularly valuable because they are often associated with equivalent functions across species. This makes them essential for transferring functional annotations from well-characterized species to less-studied ones (Koonin 2005). For bacteria, actual annotation coverage from homology search varies between 14% and 98% depending on the species (Lobb *et al.* 2020), with an average of 79% for the whole bacterial proteome. For the malaria parasite *Plasmodium falciparum*, annotation of 98% of its genes is possible with this technique (Steinbiss *et al.* 2016).

Identifying homologs based on sequence similarity relies on tools that detect conserved patterns in amino acid composition. Initially, these tools were based on the BLAST algorithm (Altschul *et al.* 1990) that uses local alignments. For instance, PSI-BLAST (Altschul 1997) is particularly adapted to pairwise comparisons for homology detection. It has been used in combination with other methods, such as Markov clustering in Li *et al.* 2003, to create OrthoMCL-DB, a large database of groups of orthologous proteins. Further, specific challenges such as detecting remote homologs—proteins sharing <30% sequence identity—have been addressed with hidden Markov models (Söding 2005, Remmert *et al.* 2012) for evolutionary-distant species. Despite the difficulties, the foundational principle that a protein’s sequence encodes all the information needed for its structure and function (Anfinsen 1973), suggests that these relationships can still be uncovered.

Recently, alignment-free methods based on machine learning have emerged as powerful alternatives for homology detection. For instance, DeepSeqProt (David and Halanych 2023) employs an encoder–decoder neural network to create clusters of orthologous proteins. Many of these methods utilize numerical representations of protein sequences, known as embeddings, instead of comparing amino acid composition directly, to encode sequence information into high-dimensional spaces. Hamamsy *et al.* (2024) use several transformer encoder layers to create a representation of the sequences in 512 dimensions and compute a cosine distance between each pair of proteins to look for remote homologs. Rosen *et al.* (2024) utilize the ESM embedding model (Rives *et al.* 2021) to create a representation of proteins in 1280 dimensions and apply a modified k-means algorithm to create clusters of similar proteins, from which homologous pairs can be extracted. SonicParanoid2 (Cosentino *et al.* 2024) employs Doc2Vec embedding to generate orthologous groups with high accuracy and speed. Overall, the recent development of large language models specific to proteins, such as SeqVec (Heinzinger *et al.* 2019), ProtT5 (Elnaggar *et al.* 2022), or ESM (Rives *et al.* 2021), promises improved representation of proteins and finer analyses.

In this study, we explore how clustering proteins embedded with a large language model can be optimized to achieve the best results in homology detection across the whole animal kingdom. Specifically, we employ a k-means clustering algorithm associated with the ESM embedding model. This pipeline is present in Rosen *et al.* (2024); however, their focus was not on homology detection, and key parameters, such as the number of clusters, were not systematically optimized. Here, we show how to optimize this solution for homology detection by increasing the number of clusters with respect to the dataset size. We compare performance at retrieving orthologous pairs and creating full orthologous groups to BLAST-based methods (OrthoMCL), SonicParanoid2, which is state-of-the-art for both tasks, and DeepSeqProt which focuses specifically on group creation. Our optimized pipeline achieves high precision in specific datasets, albeit with much-reduced sensitivity, and approaches state-of-the-art performance for group creation. The findings suggest that this approach is a promising alternative to existing methods and could benefit from further refinement to enhance its utility in homology detection.

## 2 Materials and methods

### 2.1 Orthology databases

We used OrthoMCL-DB (https://orthomcl.org/, release 6.21), which comprises 985 418 orthologous groups formed with 9 050 159 proteins distributed over 865 different species. We used a search strategy among proteins with the Taxonomy option to extract the full proteome of species of interest. We downloaded amino acid sequences as fasta files, with all information related to the organism of origin, protein description, and orthologous group assignment. We focused on the following five species complexes, described in Table 1: frog (*Xenopus tropicalis*, xtro) and zebrafish (*Danio rerio*, drer); mouse (*Mus musculus*, mmus) and human (*Homo sapiens*, hsap); 7 Apicomplexa species (*Plasmodium falciparum*, pfal; *Plasmodium berghei*, pber; *Cryptosporidium bovis*, cbov; *Cryptosporidium parvum*, cpar; *Neospora caninum*, ncan; *Toxoplasma gondii*, tgon; *Trypanosoma brucei*, tbrt); *Plasmodium falciparum* and *Plasmodium berghei*; 13 species from the tree of life (*Arabidopsis thaliana*, atha; *Caenorhabditis elegans*, cele; *Drosophila melanogaster*, dmel; *Danio rerio*, drer; *Escherichia coli*, ecol; *Homo sapiens*, hsap; *Mus musculus*, mmus; *Mycobacterium tuberculosis*, mtub; *Pan troglodytes*, ptro; *Saccharomyces cerevisiae*, scer; *Sulfolobus acidocaldarius*, saci; *Tripanosoma brucei*, tbrt; *Xenopus tropicalis*, xtro). These species were selected based on their biological relevance and evolutionary relationships. In particular,

**Table 1.** Dataset sizes and characteristics in OrthoMCL-DB.^a^

Species studied	Number of	Number of	Number of	Number of
	proteins	n:m orthologs	1:1 orthologs	OG groups
Frog-Zebrafish	61 670	120 011	3848	18 374
Mouse–human	45 087	92 063	10 289	16 659
Apicomplexa ×7	42 839	69 539	570	17 822
*Plasmodium* ×2	10 263	4789	3640	5315
Tree of life ×13	108 663	339 380	0	40 552

a We randomly select 108 663 sequences from the tree of life proteome (50% of the total) to build a large and diverse dataset. OG = orthologous.

- frog, mouse, and zebrafish represent model organisms in vertebrate developmental biology, with high-quality annotations;
- the 7 Apicomplexa species include important parasites with shared evolutionary origins but distinct host specificities;
- *Plasmodium* species are key malaria-causing pathogens with a close phylogenetic relationship; and
- the 13 species from the tree of life provide a large and diverse dataset that ensures our method is generalizable.

Within orthologous groups, we studied pairs of orthologous proteins, which are proteins coming from two different species. We distinguished between n:m orthologs (where multiple proteins from one species are orthologous to multiple proteins from another species) and 1:1 orthologs (where a single protein from one species matches only a single protein from another species). For more than two species, 1:1 orthology corresponds to an orthologous group that contains one and only one protein from each species of interest. Another type of homology is paralogy, which is when a protein is homologous to other proteins from the same species. This happens following duplications of a gene in the genome; however, this case is not necessarily associated with similarity in function, so it is excluded from our study.

We chose this database as it comes with an online platform with fasta files and homology information for proteins on a large number of species. Other tools exist, such as OrthoFinder (Emms and Kelly 2019), which is faster than OrthoMCL and more recent, but must be run from scratch on every protein dataset of interest. Here, the choice of a database is mainly to provide a reference, and the focus of this study is to compare detection methods to each other.

For supplementary analyses, we used the OrthoDB catalogue (https://www.orthodb.org/, v11) to validate orthologous groups not present in OrthoMCL-DB. OrthoDB employs a different clustering methodology for uncovering homology (Kuznetsov *et al.* 2023).

### 2.2 Machine learning pipeline for homology detection

#### 2.2.1 Protein embeddings

Following the paper by Rosen *et al.* (2024), we used the protein embedding model ESM in its second version, ESM-2 (Lin *et al.* 2023). This model has demonstrated superior performance in capturing functionally and structurally meaningful information from protein sequences. It takes as input a fasta file of amino acid sequences of proteins, and represents each of them as a *p*-dimensional numerical vector. Value of *p* depends on the choice of model, and we display in Table 2, the different versions that we used in this study and their main characteristics.

**Table 2.** Main characteristics of the different versions of the ESM-2 model used.

Version name	Number of	Embedding
	parameters	dimensions (*p*)
esm2_t6_8M_UR50D	8 M	320
esm2_t12_35M_UR50D	35 M	480
esm2_t30_150M_UR50D	150 M	640
esm2_t33_650M_UR50D	650 M	1280
esm2_t36_3B_UR50D	3 B	2560

To create the embedding, the ESM model uses a series of transformer neural networks to obtain a meaningful representation of proteins. Transformers are a type of neural network specialized in language modelling, that belong to the family of large language models. In the protein context, they are trained with the objective of predicting missing amino acids in a sequence. The larger the embedding model and the number of embedding dimensions, the more precise the representation of proteins is expected to be. ESM models have been pre-trained on about 65 million protein sequences taken from the UniRef50 dataset (Suzek *et al.* 2015), and we used the final model weights without any modification to create the embedding representations, and leave the default truncation value at 1022 amino acids for a sequence. We used by default the esm2_t36_3B_UR50D model which has 2560 embedding dimensions.

Another protein embedding model, ProtT5 (Elnaggar *et al.* 2022) is used for comparison. This model is also based on transformer networks, but uses a different architecture and training procedure than ESM.

Additionally, we explored dimensionality reduction using principal component analysis (PCA) to address computational limitations. We evaluated PCA with 20, 40, 60, 80, 100, and 120 components, comparing performance to the full embedding dimensions.

#### 2.2.2 Unsupervised clustering

After generating embeddings, and when relevant, after the PCA dimension reduction step, the data is numerical and has a fixed number of dimensions. We applied unsupervised clustering with the k-means algorithm to create orthologous groups without any prior information on the composition of the groups. This algorithm is state-of-the-art for unsupervised clustering, it is scalable to large numbers of protein sequences and orthologous groups, and it outputs distances between objects in an Euclidean space.

K-means clustering aims to separate the samples into groups called “clusters,” where the centre of each cluster is referred to as a “centroid”. Separation is done to minimize a within-cluster sum-of-squares that is based on the distance from each sample to its designated centroid. The main parameter is the number of clusters to form, and it must be specified beforehand. We varied this parameter in the study to find the optimal value depending on the data size. We used scikit-learn’s KMeans function to create clusters. We set the random seed with random_state=0 to allow reproducibility and n_init=5 to run the algorithm several times before returning the best result. All other parameters were kept to their default values. The function returns the predicted cluster of each protein as a numerical label, as well as centroid locations in the representation space. We compared predicted cluster compositions to the true orthologous groups.

#### 2.2.3 Extraction of orthologous pairs

To identify n:m orthologs within clusters, we used two approaches:

- Naive pairing (“all pairs”): all pairwise combinations of proteins from different species within the same cluster are considered orthologs.
- Distance-based pairing (“top pairs”): for each cluster, the closest protein to the cluster centroid and the next closest from a different species are selected.

**Figure 1.**
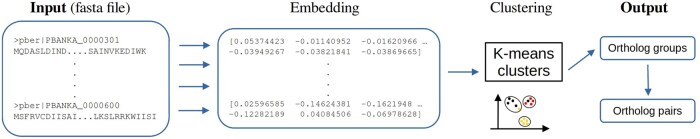
Pipeline of the method proposed in this article.

**Figure 2.**
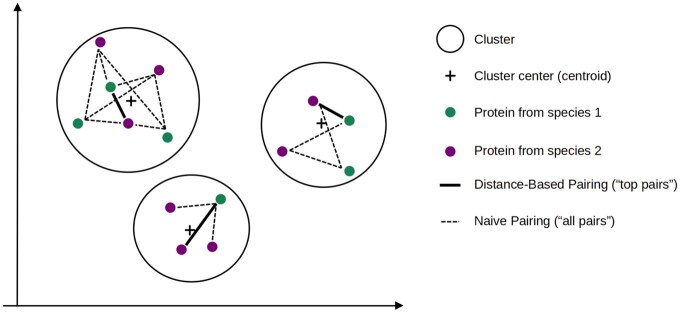
Highlight of the procedure to select orthologous pairs from orthologous groups (clusters). Naive Pairing includes the Distance-Based Pairing results. This procedure is also valid for more than 2 species.

Both approaches exclude clusters containing only proteins from the same species (to avoid paralogs) or those of size 1. For detecting 1:1 orthologs, we restricted the analysis to clusters whose size is equal to the number of species studied, and referred to our approach only as “k-means.” Predicted orthologs were matched against OrthoMCL-DB. Figure 1 presents the pipeline of the proposed method, and Fig. 2 is a detailed illustration of the procedure to select orthologous pairs from orthologous groups (clusters).

Distance-based selection is also applied in the article by Rosen *et al.* (2024). Their method uses the same embedding model, and they also select proteins by decreasing distance from the centroid in the Euclidean space. However, the full clustering pipeline used by these authors is different from a mere k-means, as the final distances from the centroids are modified to achieve a wider range of objectives, including homology search but also differential expression, cell-type label transfers. It also takes into account additional information on gene counts as input data, which we did not use here.

### 2.3 Other methods for homology detection

We compared our pipeline to state-of-the-art methods for homology detection from the literature: SonicParanoid2 (Cosentino *et al.* 2024) and DeepSeqProt (David and Halanych 2023).

SonicParanoid2 uses two models in parallel to infer orthologous groups: a domain-based orthology inference algorithm and a graph-based model with an AdaBoost classifier that uses the embedding model Doc2Vec (Mikolov *et al.* 2013) to generate numerical representations of the proteins. Results are then merged with a Markov cluster algorithm to create clusters of orthologs, and cluster compositions are returned by species. Proteins that are not in a cluster are put into a separate list that we handled as clusters of size one. SonicParanoid2 is optimized for speed, efficiency, and accuracy compared to similar machine learning methods.

DeepSeqProt uses one-hot encoding representation instead of an embedding model. This technique is adapted to categorical data and transforms amino acid protein sequences into a numerical input of binary covariates, with one binary covariate for each amino acid at each position in the sequence. DeepSeqProt then applies unsupervised clustering in the form of an encoder–decoder neural network, with a vector quantization unit in the middle to associate each sequence to a single cluster, and returns cluster compositions as the final output. In practice, this method tends to gather proteins into large clusters and uses <100 clusters in total. It focuses mainly on grouping together similar sequences, and less on putting apart different protein families. Since we do not have species-to-species information with this method, nor a distance-type information, we extracted orthologs by taking all pairwise combinations within the clusters, which yielded poor precision values, but high sensitivity.

For both methods, we kept all parameters to their default values. Further, in the 1:1 orthology case, we focused only on groups of size equal to the number of species of interest in the datasets (two or seven).

For further validations of orthologous pairs, we also computed their alignment scores with PSI-BLAST using the NcbiblastpCommandline function from Python’s Bio package.

### 2.4 Performance metrics

A number of metrics were used to assess the performance of the different methods at finding orthologous pairs and creating full orthologous groups. Although this is not necessarily true, we decided to use OrthoMCL-DB as the ground truth.

For pairwise orthology, we computed the precision and the sensitivity of the prediction:


$$\begin{array}{c} \text{precision}=\frac{\text{number}\;\text{of}\;\text{correct}\;\text{orthologs}}{\text{total}\;\text{number}\;\text{of}\;\text{orthologs}\;\text{retrieved}}\\ \text{sensitivity}=\frac{\text{number}\;\text{of}\;\text{correct}\;\text{orthologs}}{\text{true}\;\text{number}\;\text{of}\;\text{orthologs}\;\text{in}\;\text{OrthoMCL-DB}} \end{array}$$

They give complementary information: precision tells how reliable it is to use clusters this way to retrieve orthologs, and sensitivity informs how many orthologs from the total we can retrieve.

We also compute the F1-score. In our case, the model only outputs positive predictions, so the F1-score corresponds to the harmonic mean between precision and sensitivity:


$$F1-\text{score}=\frac{2}{{\text{precision}}^{-1}+{\text{sensitivity}}^{-1}}$$

For group creation, we used three different metrics. The first is family completeness, introduced in the DeepSeqProt paper (David and Halanych 2023) and defined as:


$$\frac{\sum_{i=1}^{I}\max(\{y_{i,1},\ldots ,y_{i,n}\})}{T},$$

where *I* is the total number of true orthologous groups, *y* is the number of representatives from orthologous group *i* across *n* clusters, and *T* is the total number of protein sequences in the dataset. Family completeness represents the greatest fraction of sequences from the same group that are categorized together. It falls between 0 and 1 and values close to 1 are best.

The second is the adjusted mutual information (AMI) (Vinh *et al.* 2010), which measures how well clustering was done compared to a randomly generated clustering. It takes values between 0 and 1, a value of 0 means that all members of a true orthologous group belong to different clusters, and a value of 1 means that all members of an orthologous group belong to the same cluster and each cluster contains only a single orthologous group. The AMI is agnostic to the number of clusters thus enables comparison of different methods. We computed AMI with the function adjusted_mutual_info_score from scikit-learn.

The third metric is the percentage of exact matches, defined as the percentage of clusters that correspond exactly to an orthologous group from OrthoMCL-DB. It means that these clusters contain all sequences from a true orthologous group, and only these ones. Since a single protein falling in the wrong cluster yields two flawed clusters, this metric is more conservative than the two others at evaluating the creation of whole sets of objects.

## 3 Implementation

We used Python 3.10.12 for the implementation. Version numbers for all packages needed is present in Table 3. They are required to run ESM to create the embeddings and cluster proteins with the k-means algorithm. They can be installed all at once with a $pip install command inside a virtual environment. We used a graphics processing unit (GPU) to speed up protein embedding. For the ESM models with up to 650 M parameters, an Nvidia RTX A1000 GPU with 6 GB of memory was sufficient. The largest model with 3B parameters was run on an Nvidia A40 GPU with 48 GB of memory. Cluster creation with k-means did not require GPU usage and could be run on a regular laptop. We used up to 20 cores in parallel to speed up computation. For creating figures, we used the R packages ggplot2 (Wickham 2016, https://ggplot2.tidyverse.org/) and ggbreak (Xu *et al.* 2021).

**Table 3.** Python packages used in this study.

Package name	Version number
biopython	1.79
deepspeed	0.5.9
dm-tree	0.1.6
fair-esm	2.0.0
matplotlib	3.7.4
ml-collections	0.1.0
numpy	1.21.2
pandas	1.4.2
requests	2.26.0
scikit-learn	1.3.2
scipy	1.7.2
seaborn	0.13.1
tqdm	4.62.2
typing-extensions	4.11.0
pyqt5	5.15.10
pytorch_lightning	1.5.10
wandb	0.12.21

## 4 Results

We first studied the performance of k-means clustering on a particular dataset according to hyperparameter settings, namely the number of clusters, the use or not of PCA dimension reduction, and the choice of the embedding model. We then compared the performance of k-means clustering, SonicParanoid2, and DeepSeqProt at retrieving orthologous pairs and full orthologous groups. Finally, we performed a more detailed analysis of orthologs between *P. berghei* and *P. falciparum*.

### 4.1 Performance according to model parameters

#### 4.1.1 Number of clusters

The main parameter in k-means clustering is the number of clusters, k. We evaluated its impact on performance using the *P. falciparum* and *P. berghei* datasets.

**Figure 3.**
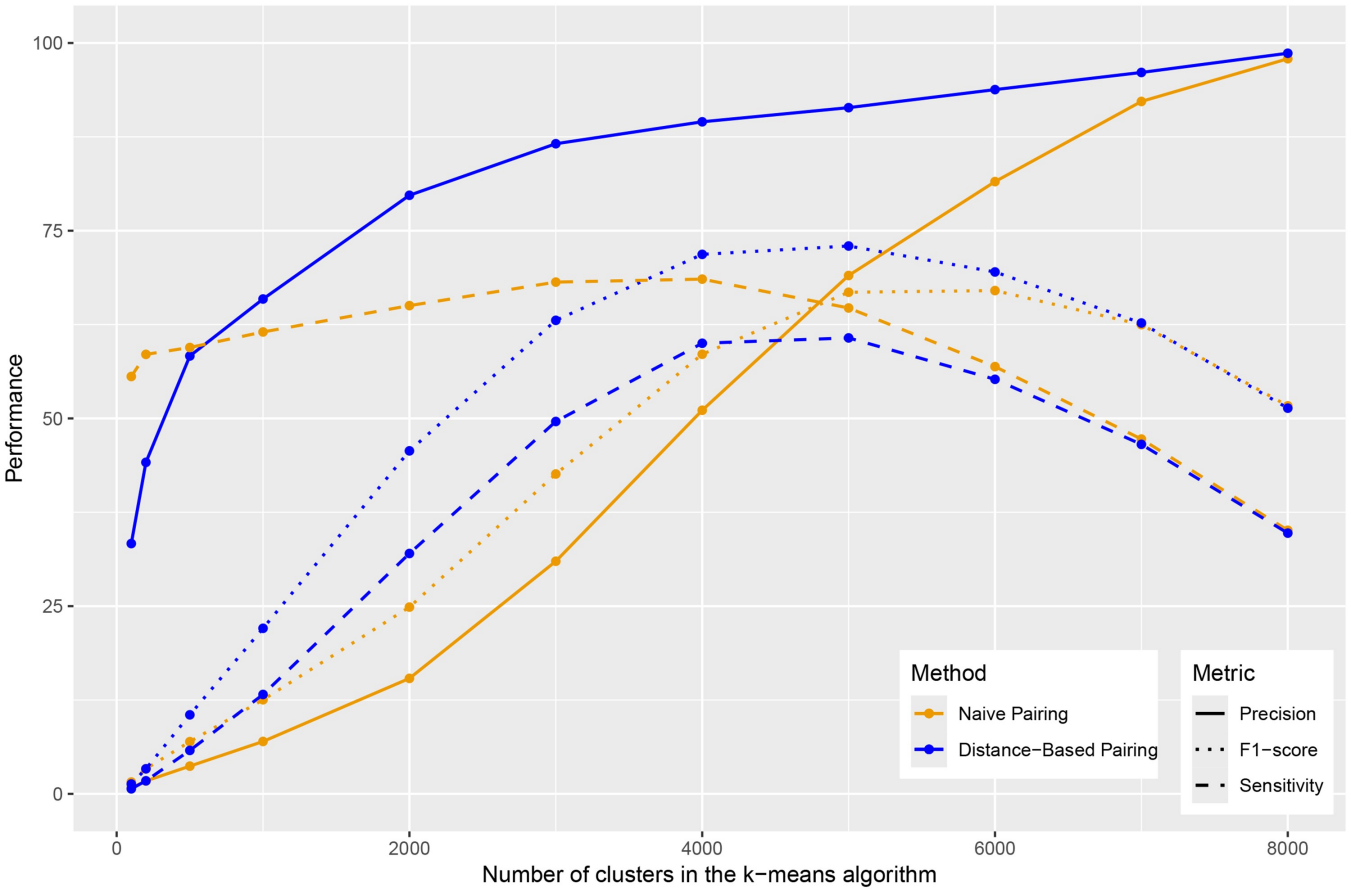
Performance for n:m ortholog detection with respect to the number of clusters. Clustering of 10 263 protein sequences from *P. falciparum* and *P. berghei*.

For retrieving n:m orthologs, precision always increased with higher *k* values (Fig. 3), reaching nearly 100% at the highest cluster counts. Sensitivity was consistently below 75% for this dataset. In particular, we revealed an optimum when k is close to half the size of the dataset: 5000 clusters for Distance-Based Pairing and 4000 clusters for Naive Pairing. We observed a similar optimum in sensitivity with respect to data size for other datasets, as well as for sub-selections of a given dataset (see Fig. 1, available as supplementary data at *Bioinformatics* online). Hence, we set a number of clusters equal to half the size of the dataset (rounded down for odd numbers) in Section Comparing performance on different datasets. This value balances sensitivity and precision while simplifying parameter selection.

Low performance for small values of k-means that the clustering algorithm might not have converged, while for high values of k, sensitivity starts to decrease. We explain this in Fig. 3, available as supplementary data at *Bioinformatics* online, where we count the number of cluster of each size, for different values of *k*, on the *P. falciparum* and *P. berghei* datasets. We find that for low values of *k*, small and large clusters occur in similar proportions, while for high values of *k*, there are mainly small clusters of size 1 or 2. For k=8000, the highest value tested, we found 6113 clusters of size 1, 1773 clusters of size 2, and 114 clusters of greater sizes. In particular, clusters of size 1 become prevalent as *k* increases. As these clusters do not contain any homolog pairs, this naturally decreases the total number of homologs retrieved, and the sensitivity. Regarding precision, when k increases, the number of clusters of sizes >1 decreases, so these clusters become more selective.

Sensitivity was higher when using Naive Pairing compared to Distance-Based Pairing, which is expected, but then the precision was much lower, and reached values above 90% only for large *k*, k=7000 and k=8000. Both this and the sensitivity cap are issues at the moment, highlighting limitations of k-means clustering. When the number of clusters increased, they tended to be smaller in size, hence both pairing approaches yielded closer results.

In terms of the F1-score, we also observed an optimum when k is close to half the number of sequences in the dataset, for the *P. falciparum* and *P. berghei* datasets and the frog-zebrafish dataset (Fig. 3 and Fig. 1, available as supplementary data at *Bioinformatics* online).

**Figure 4.**
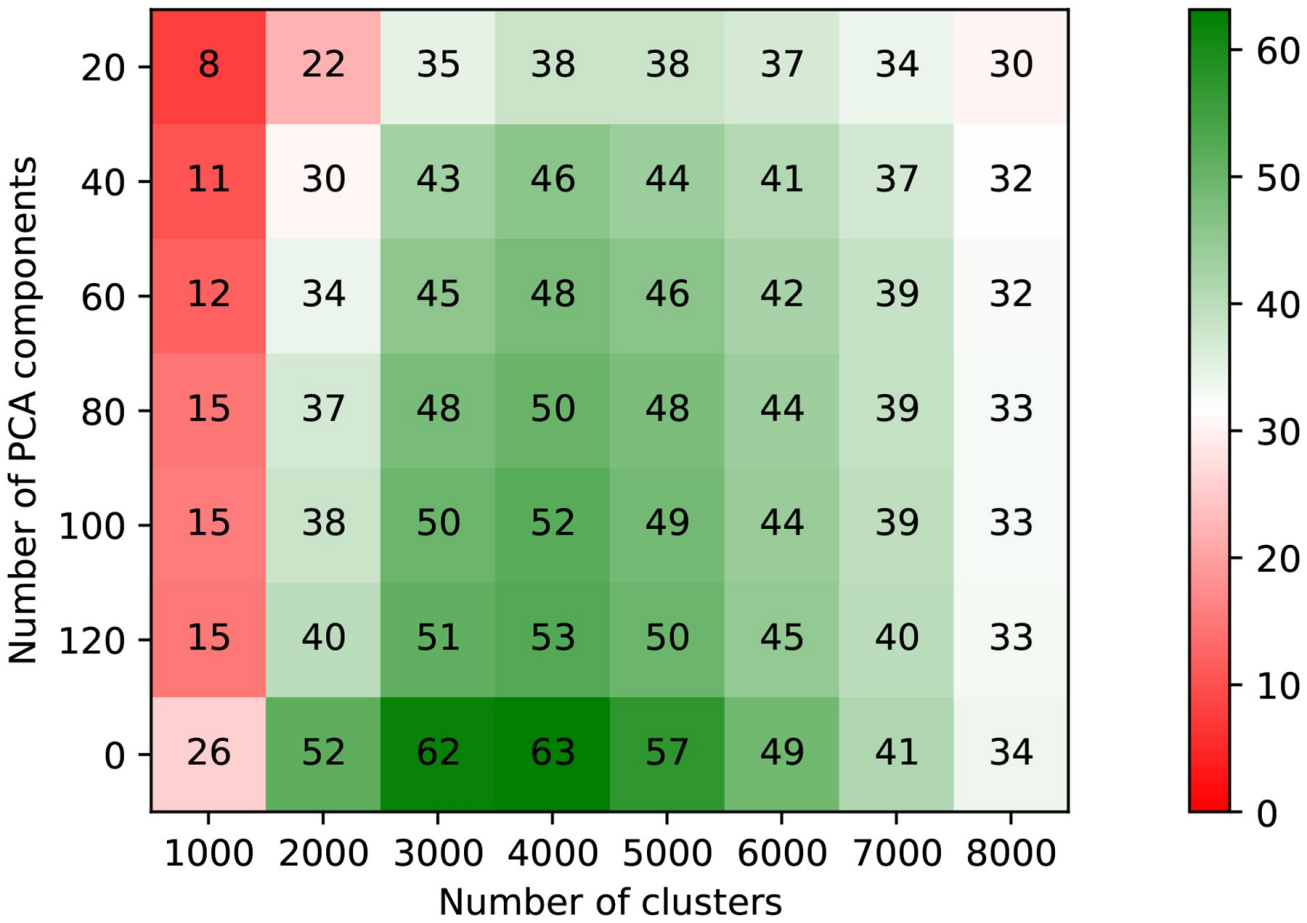
Percentage of clusters that correspond exactly to a true group from OrthoMCL-DB. Clustering of 10 263 protein sequences from *P. falciparum* and *P. berghei*. Value 0 for PCA components means that we do not use dimension reduction.

Regarding group creation, we also observed optimal performance when *k* was close to half the size of the dataset. For the *Plasmodium* data, the highest percentage of clusters that correspond exactly to an orthologous group was found for k=4000 (Fig. 4) and equalled 63%. This reinforced the previous conclusion of using the dataset size to set the number of clusters. Further, we showcased the impact of using PCA dimension reduction before clustering the data. From a default embedding with 2560 dimensions, performance dropped by about 10 percentage points when using 120 PCA components and decreased further when using stronger dimension reduction.

#### 4.1.2 Size of the embedding model

**Figure 5.**
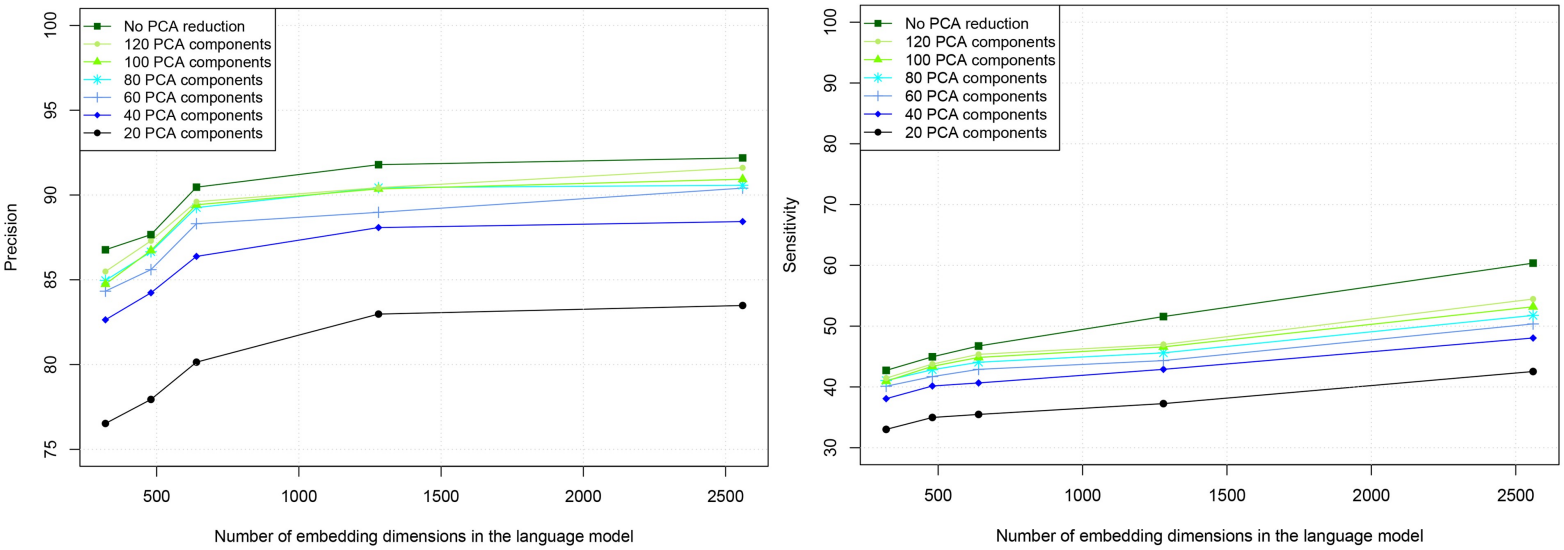
Performance at retrieving n:m orthologs. Left: precision. Right: sensitivity. Clustering of 10 263 protein sequences from *P. falciparum* and *P. berghei*, using 5131 clusters and Distance-Based Pairing.

As demonstrated above with the use of PCA dimension reduction, the precision of the embedding representation directly impacts the efficiency of clustering and the performance at homology detection. Several sub-models exist in the ESM framework (see Table 2), each with a different number of embedding dimensions. In this section, we studied how performance evolves with the size of the embedding representation. The initial assumption is that the higher the dimension is, the more precise the model should be. We verified this assumption with respect to precision and sensitivity at n:m ortholog detection in Fig. 5.

We found that sensitivity increased almost linearly with the number of embedding dimensions, and that there was a significant improvement in using all embedding dimensions compared to applying PCA reduction. However, precision increased and then reached a plateau above 90% from 1280 embedding dimensions, and performance on the full data and on the data with 80 or more PCA components was very close, meaning that larger models will not necessarily yield better precision.

Choosing a larger embedding model comes with higher hardware requirements. As shown in Table 2, doubling the number of embedding dimensions corresponds to multiplying the number of parameters in the model by a factor of approximately 5, posing challenges for memory and computational efficiency.

### 4.2 Comparing performance on different datasets

We then compared k-means clustering, SonicParanoid2, and DeepSeqProt using full proteomes from selected species, with k set to half the dataset size, as detailed in Section Number of clusters. Within all datasets except the Tree of Life one, species have comparable numbers of proteins, so there is no particular imbalance. For the latter dataset, we have differences in proportion up to 1 to 10.

For retrieving pairs of orthologous proteins, we achieved the best precision with Distance-Based Pairing, with values above 90% for all groups of species studied (Table 4). Naive Pairing reached over 90% precision in two datasets only, frog-zebrafish and mouse–human, and performed worse than SonicParanoid2 in the Apicomplexa ×7 and the *Plasmodium* ×2 datasets. Both k-means approaches had lower sensitivity than SonicParanoid2 and DeepSeqProt.

**Table 4.** Performance for detecting n:m orthologs.^a^

	Precision				Sensitivity				F1-score			
	k-means	k-means	SP2	DSP	k-means	k-means	SP2	DSP	k-means	k-means	SP2	DSP
	(top pairs)	(all pairs)			(top pairs)	(all pairs)			(top pairs)	(all pairs)		
Frog-Zebrafish	**94.8**	93.1	78.2	0.111	4.70	17.3	30.9	**57.7**	8.96	29.2	**44.3**	0.222
Mouse–human	**98.3**	92.0	86.7	0.264	13.9	21.6	24.4	**75.2**	24.4	35.0	**38.1**	0.526
Apicomplexa ×7	**90.5**	63.7	75.8	0.080	13.6	20.9	**69.1**	30.3	23.6	31.5	**72.3**	0.160
*Plasmodium* ×2	**92.2**	72.3	89.6	0.157	60.4	64.0	**84.6**	49.3	73.0	67.9	**87.0**	0.313
Tree of life ×13	**91.4**	80.1	21.0	0.055	3.13	19.2	**69.0**	53.8	6.05	31.0	**32.2**	0.110

a Top pairs = Distance-Based Pairing; All pairs = Naive Pairing; SP2 = SonicParanoid2; DSP = DeepSeqProt. Bold values indicate the best performing method for each metric and each dataset.

In four out of five datasets, SonicParanoid2 reached precision values between 75% and 90%, and DeepSeqProt had very low precision, under 1%, because it returned much larger groups of proteins than the ground truth and, thus, more false positives. For sensitivity, best results were achieved by DeepSeqProt in frog-zebrafish and mouse–human datasets, and by SonicParanoid2 for the other datasets. SonicParanoid2 achieves best results in terms of F1-score.

For 1:1 ortholog retrieval, k-means clustering and SonicParanoid2 had comparable precision (within 6 percentage points), with k-means outperforming SonicParanoid2 in most datasets except frog-zebrafish (Table 5). However, SonicParanoid2 had significantly higher sensitivity, reaching values between 82.1% and 99.2%, and the best F1-score values. K-means F1-score is close to the best in 2 datasets out of 4, namely the mouse–human and the Plasmodium ones. DeepSeqProt failed to retrieve meaningful 1:1 orthologs.

**Table 5.** Performance for detecting 1:1 orthologs.^a^

	Precision			Sensitivity			F1-score		
	k-means	SP2	DSP	k-means	SP2	DSP	k-means	SP2	DSP
F-Z	58.6	**63.4**	0.0	39.9	**85.2**	0.0	47.5	**72.7**	∅
M-H	**71.3**	66.8	∅	75.0	**98.1**	0.0	73.1	**79.5**	∅
A7	**80.0**	78.4	∅	1.4	**82.1**	0.0	2.8	**80.2**	∅
P2	**87.1**	81.5	0.0	67.8	**99.2**	0.0	76.2	**89.5**	∅

a F-Z = Frog-Zebrafish; M-H = Mouse–human; A7, Apicomplexa ×7; P2 = Plasmodium ×2; SP2 = SonicParanoid2; DSP = DeepSeqProt; A ∅ symbol means that the method has not retrieved any 1:1 ortholog. The Tree of Life dataset has no 1:1 orthologs according to our definition and is thus not included. Bold values indicate the best performing method for each metric and each dataset.

Altogether, k-means clustering is a conservative alternative to state-of-the-art methods for retrieving both n:m and 1:1 orthologs. It reaches a precision above 90%, better than state-of-the-art methods, ensuring high reliability of the results. However, it is clearly outperformed by other methods in terms of sensitivity. The performance of SonicParanoid2 is even and it shows good performance in terms of precision, sensitivity, and F1-score. DeepSeqProt has high sensitivity but otherwise struggles at species-to-species comparison, especially on 1:1 orthology.

For creating full orthologous groups, k-means clustering came close to state-of-the-art results (Table 6). It ranked second or third in terms of family completeness. For the AMI, SonicParanoid2 was better overall, but k-means yielded the best value for the mouse–human dataset. For the percentage of exact orthologous groups created, k-means clustering was again behind SonicParanoid2, but scored above 30% in all datasets. This task is particularly difficult, and DeepSeqProt failed to create any group correctly, so this is still a promising performance of k-means clustering.

**Table 6.** Performance for orthologous group creation.^a^

	Family			Adjusted Mutual			Percentage of		
	Completeness			Information			exact matches		
	k-means	SP2	DSP	k-means	SP2	DSP	k-means	SP2	DSP
Frog-Zebrafish	0.626	0.708	**0.757**	0.679	**0.694**	0.215	31.6	**39.5**	0.0
Mouse–human	0.753	0.764	**0.810**	**0.744**	0.707	0.189	45.9	**55.5**	0.0
Apicomplexa ×7	0.637	**0.864**	0.593	0.497	**0.796**	0.107	30.6	**63.0**	0.0
*Plasmodium* ×2	0.828	**0.896**	0.750	0.623	**0.761**	0.113	56.1	**72.2**	0.0
Tree of life ×13	0.673	**0.878**	0.694	0.620	**0.715**	0.156	34.8	**55.5**	0.0

a SP2 = SonicParanoid2; DSP = DeepSeqProt. Bold values indicate the best performing method for each metric and each dataset.

In general, DeepSeqProt tended to output a relatively small number of large groups, typically around 50, and thus failed to match the exact composition of the thousands of orthologous groups in the different datasets. We emphasize that DeepSeqProt was developed specifically to optimize the Family Completeness metric, but not necessarily the other metrics, which could explain the high differences in performance compared to k-means and SonicParanoid2.

We tested our pipeline, using the same clustering procedure, but with ProtT5 embedding instead of ESM. On the human-mouse dataset, we obtained a precision of 98.5% with top pairs selection for n:m orthologs, and 56.2% of clusters corresponded exactly to an ortholog group, better than the other methods tested. The full results on this dataset are available in Table 1, available as supplementary data at *Bioinformatics* online. This confirms the efficiency of the embedding and clustering pipeline for identifying homologs in protein sequence datasets over other pipelines. It further highlights that protein modelling is an ongoing field worth exploring further.

### 4.3 Computation time

A full analysis for the proposed pipeline consists of three steps: embedding the data, clustering, and analysing the clusters. On a laptop with GPU enabled and 20 CPU cores, for the dataset with 42 839 protein sequences, the embedding into 1280 dimensions took 4 h 15 min, clustering using 21 419 clusters took 2 h 40 min (and only 21 min when using PCA dimension reduction with 120 components), and analysing the final clusters took a few minutes. Embedding into 2560 dimensions took several days, and the ESM software is already optimized, so this part can hardly be sped up. For comparison, SonicParanoid2 and DeepSeqProt have been optimized for speed efficiency and processed the same dataset in <10 min.

### 4.4 Homology detection in *Plasmodium* data

**Figure 6.**
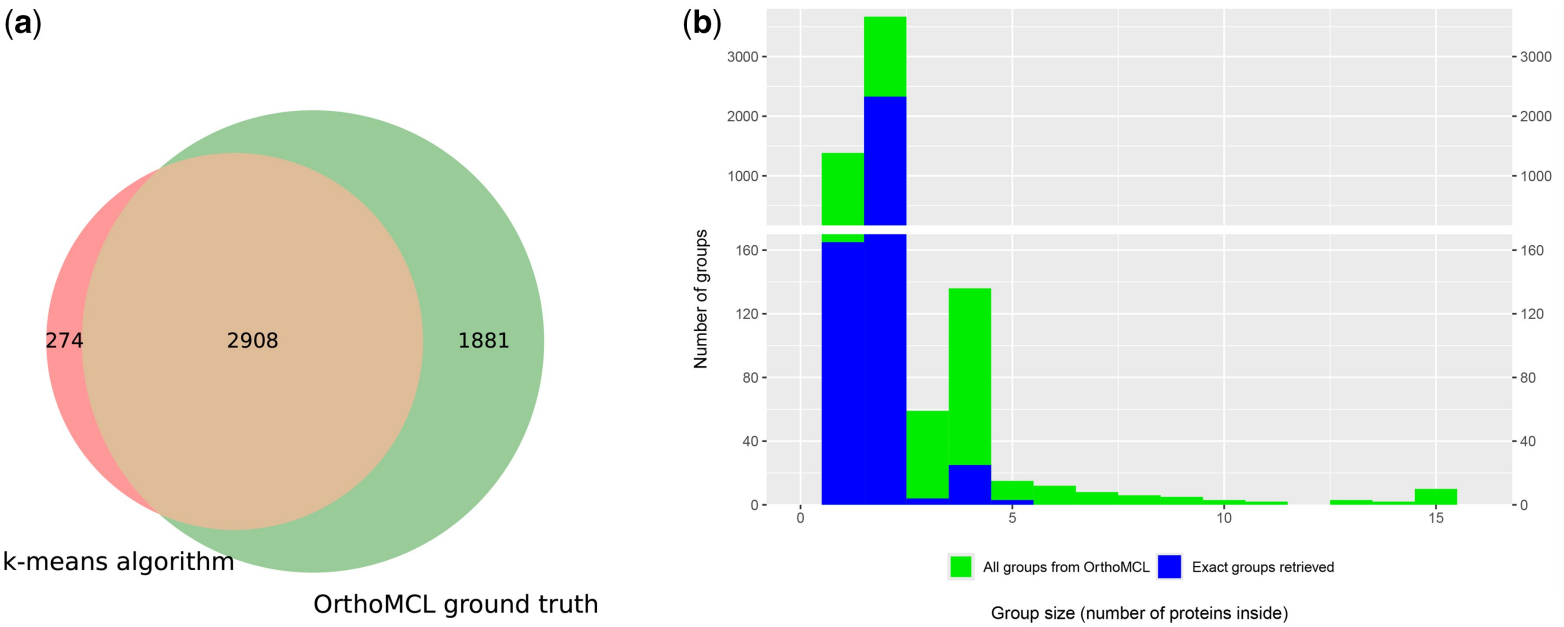
Performance at clustering 10 263 protein sequences from *P. falciparum* and *P. berghei*. (a) Retrieving n:m orthologs with Distance-Based Pairing. K-means clustering with 5000 clusters. False positives in the left area (red), missed orthologs in the right area (green). (b) Retrieving full orthologous groups by group size, k-means clustering with 4000 clusters. 63% of clusters match a group exactly. We collapsed 10 groups of size ≥15 to 15.

We conducted a detailed analysis of the two *Plasmodium* species using optimal settings: 5000 clusters for n:m ortholog detection and 4000 clusters for orthologous group creation. We selected n:m orthologs with Distance-Based Pairing, which gave a precision of 91% and sensitivity of 61% (Fig. 6a). Group creation performance varied by size, with the best results observed for 1:1 orthologs (groups of size 2) (Fig. 6b).

For n:m orthologs, there were 274 putative False Positives with our proposed method in Fig. 6a. They correspond to protein pairs detected as orthologs but that are in different groups in OrthoMCL-DB. We investigated these further to verify whether they might correspond to newly discovered orthologs. First, we matched them against another orthology database, OrthoDB, where we found 126 of them. Of the remaining 148 pairs, 26 had significant PSI-BLAST alignment scores (E-value $<10^{-3}$), indicating potential novel orthologs. For example, PF3D7_1449200 and PBANKA_1312900 shared 60% sequence identity and conserved synteny, warranting further validation through experiments.

## 5 Discussion

Large language models have transformed numerous domains of scientific research and practical applications. Here, we applied these models to understand the evolution of proteins, presenting a straightforward framework for detecting n:m orthologs and forming full orthologous groups. By embedding proteins using the ESM language model and employing k-means clustering, we achieved optimal precision and good performance at orthologous group creation. However, our approach showed limitations in sensitivity and computational efficiency compared to state-of-the-art methods SonicParanoid2 and DeepSeqProt.

From k-means clusters, it was possible to retrieve orthologs by studying either the proteins closest to a cluster’s centre or all pairwise combinations within a cluster. The first approach yielded optimal precision results, exceeding 90% across all datasets, but at the cost of lower sensitivity and thus lower values in the F1-score. The second approach achieved better sensitivity but at the expense of a drop in precision. While k-means clustering was competitive with existing methods for full group creation, it did not surpass them. Moreover, performance varied with dataset characteristics, such as group sizes and evolutionary divergence among species, highlighting potential areas for further investigation.

Missed or incorrectly predicted orthologs arise from either inaccurate positioning of proteins in the embedding space or errors in cluster formation with the k-means algorithm. It is possible to improve embedding representations for proteins up to the constraints posed by overfitting. Regarding clustering, fine-tuning the number of clusters is recommended to obtain the best performance; however, a difference from OrthoMCL-DB will always remain since this database uses another clustering algorithm.

Rives *et al.* (2021) showed that orthologous proteins cluster together in the embedding space, and the k-means algorithm seems to be well-adapted to retrieve these clusters of orthologs. In addition, this algorithm is more straightforward than SonicParanoid2, which uses two clustering models in parallel and merges the results, or DeepSeqProt, which uses an encoder–decoder network of greater complexity. Other unsupervised clustering algorithms exist, for instance, hierarchical clustering or DBSCAN (Schubert *et al.* 2017), and some can be faster than k-means clustering, so that they could be of interest. However, they handle distances between objects and cluster centres differently; some even do not use Euclidean distances, so we have not used them in this study.

Going further, tools like SATURN (Rosen *et al.* 2024) make use of large language models for integrating and analysing gene expression data. In particular, for the homology detection problem, we showed that increasing the number of clusters in SATURN method is a way of improving its performance (Fig. 2, available as supplementary data at *Bioinformatics* online). This way, our methodology can enhance existing tools for data integration.

The field of large language models for protein representation is evolving rapidly. Recently, there have been attempts to include structure information in the models (Blaabjerg *et al.* 2024) or to develop lighter and faster embedding frameworks (Shang *et al.* 2024). While we conducted this study, a third version of the ESM model was also released (Hayes *et al.* 2025). The ability of such models to fathom evolutionary information makes them excellent candidates to improve over previous methods not necessarily based on machine learning. We believe that such embedding and clustering pipelines should be systematically tested against traditional methods on the task of homology detection to obtain the best possible performance, especially in the cases where these latter methods have shown their limits: distant species, remote homologs. We provide guidance to set up parameters in the k-means algorithm, so that it can be applied to a wide range of protein datasets. We hope that this work serves as a basis for further applications of machine learning in the field of homology detection. Additionally, we recommend that researchers stay up-to-date with the development of new protein embedding models, as new and better tools are likely to emerge.

## Supplementary Material

btaf472_Supplementary_Data
