# Supplementary material for: Exploring homology detection via k-means clustering of proteins embedded with a large language model
Source: Bioinformatics. 2025 Aug 26;41(10):btaf472. doi: 10.1093/bioinformatics/btaf472 (PMC12517335; doi:10.1093/bioinformatics/btaf472)
Supplement: btaf472_Supplementary_Data [file btaf472_supplementary_data.pdf]

Supplementary materials for

Exploring homology detection via k-means clustering of proteins embedded with a large language model

Thomas Minotto[1,2], Antoine Claessens[1] and Thomas D. Otto[1,3]

[1]LPHI, CNRS, University of Montpellier, 34095 Montpellier, France

[2]IMAG, CNRS, University of Montpellier, 34095 Montpellier, France

[3]School of Infection and Immunity, University of Glasgow, Glasgow G12 8QQ, UK

This PDF file includes:

Supplementary Figure 1

Supplementary Figure 2

Supplementary Figure 3

Supplementary Table 1

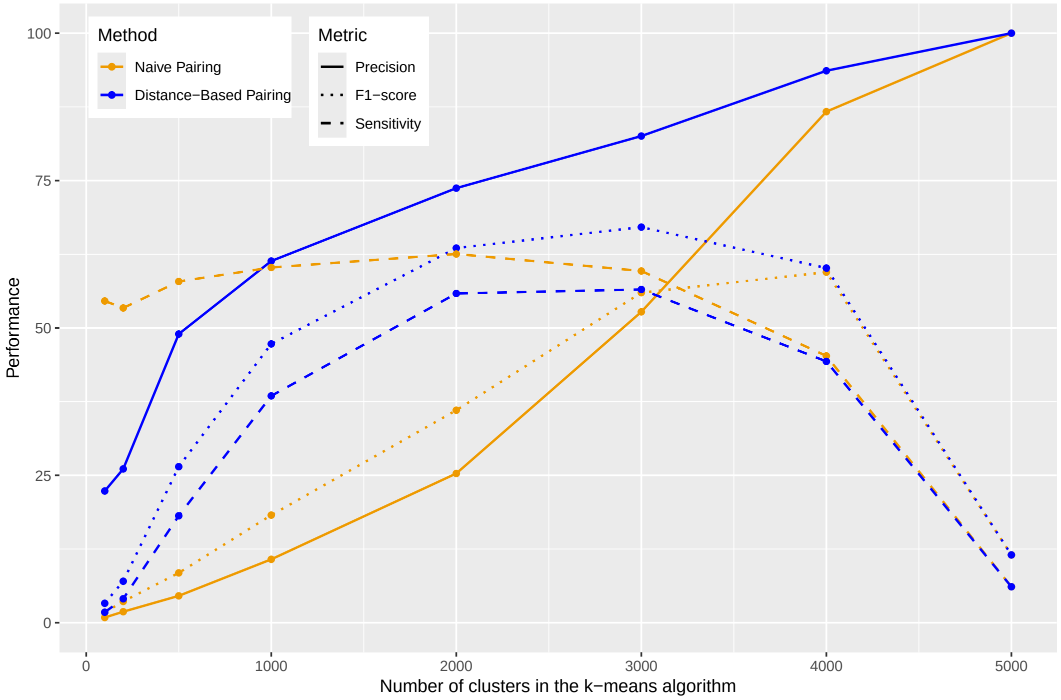

(a) Dataset of *P. berghei* and *P. falciparum*, reducing the input data by 50%, totalling 5,131 sequences

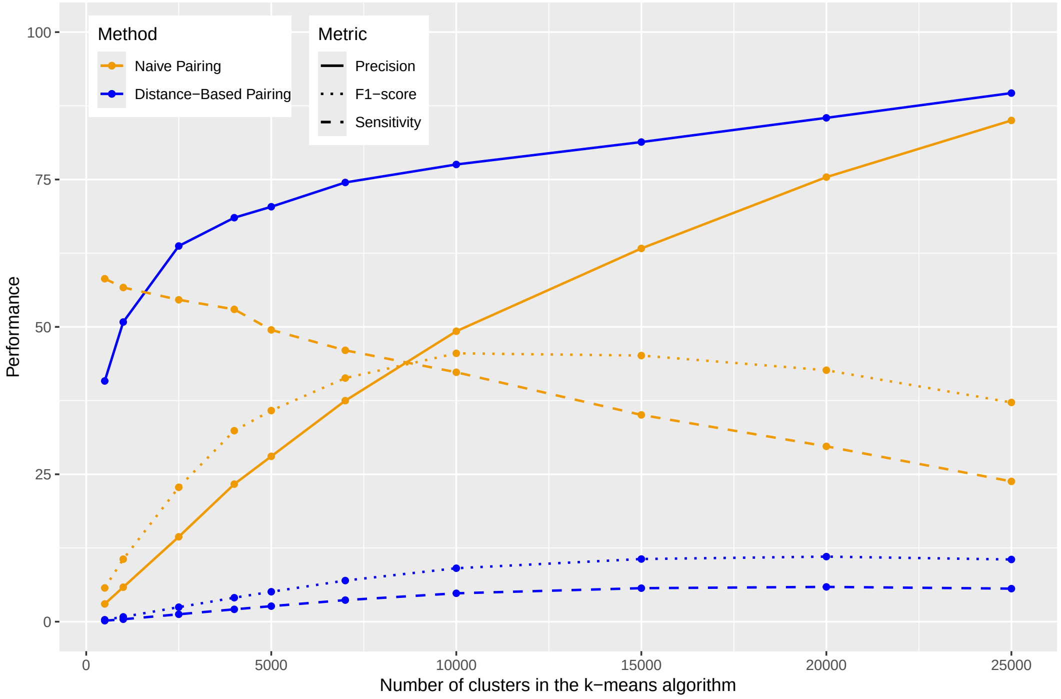

(b) Frog and zebrafish dataset, with 61,670 sequences

Figure 1: Exploration of the number of clusters needed for optimal performance. Performance for detecting n:m orthologs with an embedding using 650M parameters. We observe an optimum for sensitivity with the Distance-Based Pairing method, that is close to half the number of sequences in the dataset. The optimum with Naive Pairing varies by dataset. Precision always increases with the number of clusters.

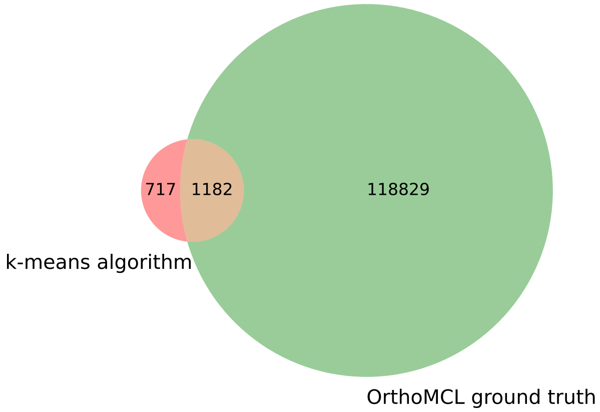

(a) Retrieving n:m orthologs with 2,000 clusters

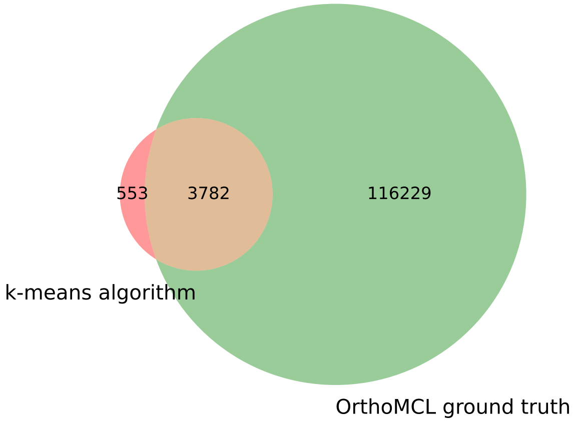

(b) Retrieving n:m orthologs with 20,000 clusters

Figure 2: Number of clusters from the k-means algorithm that yield correct orthologs (orange), false positives (red) and missed orthologs (green), selected with Distance-Based Pairing. Green parts are downscaled by a factor 5, for displaying purpose. Unsupervised clustering of 61,670 protein sequences from frog and zebrafish, embedded with 650M parameters. By increasing the number of clusters, we are able to improve both precision and sensitivity of the prediction compared to the settings with 2,000 clusters used in the SATURN paper.

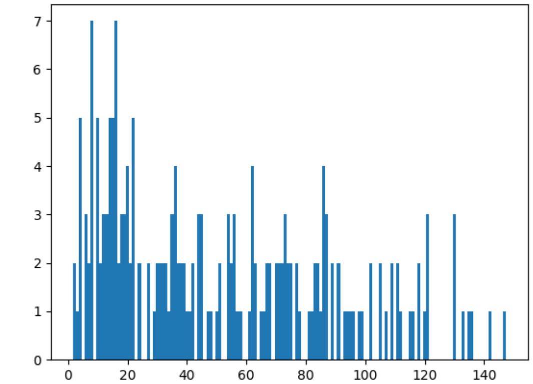

(a) Clustering with $k = 200$ clusters

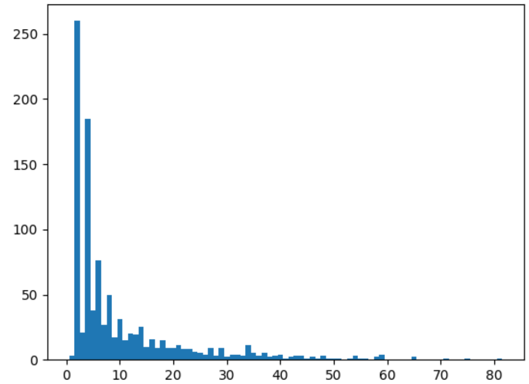

(b) Clustering with $k = 1000$ clusters

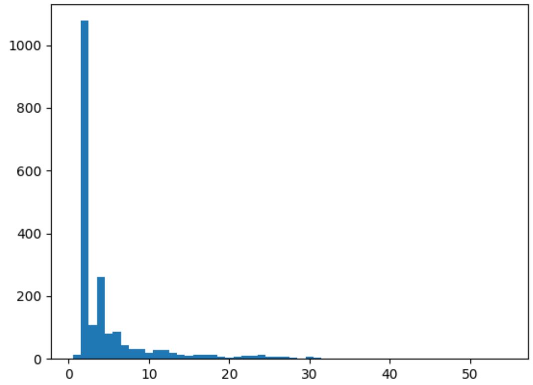

(c) Clustering with $k = 2000$ clusters

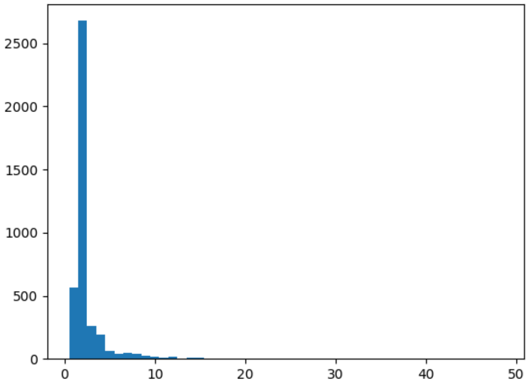

(d) Clustering with $k = 4000$ clusters

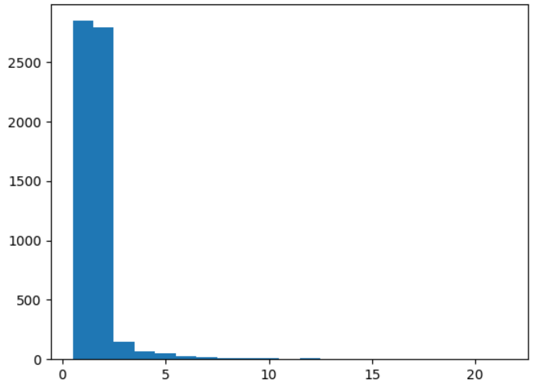

(e) Clustering with $k = 6000$ clusters

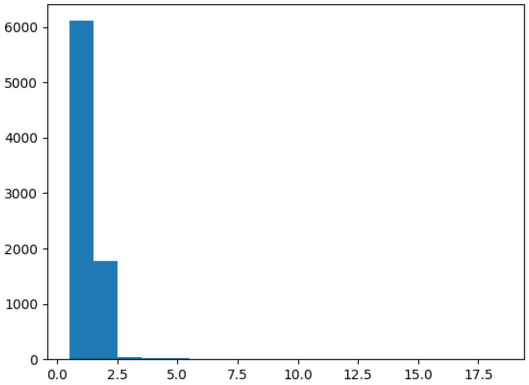

(f) Clustering with $k = 8000$ clusters

Figure 3: Clustering of 10,263 protein sequences from *P. falciparum* and *P. berghei*. Histogram of counts of clusters according to their sizes, for different values of the total number of clusters k used in the k-means algorithm. Reading: for a low value of k, we find small and large clusters in similar proportions, while for a high value of k, we mainly find small clusters of size 1 or 2.

n:m orthologs (top pairs)			n:m orthologs (all pairs)			1:1 orthologs		
Precision	Sensitivity	F1-score	Precision	Sensitivity	F1-score	Precision	Sensitivity	F1-score
**98.5**	12.8	22.7	84.4	20.0	32.3	**77.5**	80.4	78.9

(a) Orthology detection

Family Completeness	Adjusted Mutual Information	Percentage of exact matches
**0.812**	**0.750**	**56.2**

(b) Group level metrics

Table 1: Homology detection performance on the human-mouse dataset, using the embedding and clustering pipeline with the ProtT5 model for embedding, and 22,543 clusters in the k-means algorithm. Numbers in bold indicate better performance than any of the other tested methods, ESM2, SonicParanoid2 and DeepSeqProt.
